# mRNA-COVID19 Vaccination Can Be Considered Safe and Tolerable for Frail Patients

**DOI:** 10.3389/fonc.2022.855723

**Published:** 2022-03-17

**Authors:** Maria Teresa Lupo-Stanghellini, Serena Di Cosimo, Massimo Costantini, Sara Monti, Renato Mantegazza, Alberto Mantovani, Carlo Salvarani, Pier Luigi Zinzani, Matilde Inglese, Fabio Ciceri, Giovanni Apolone, Gennaro Ciliberto, Fausto Baldanti, Aldo Morrone, Valentina Sinno, Franco Locatelli, Stefania Notari, Elena Turola, Diana Giannarelli, Nicola Silvestris

**Affiliations:** ^1^ Hematology and Bone Marrow Transplantation Unit, IRCCS San Raffaele Scientific Institute, Milano, Italy; ^2^ Biomarkers Unit, Department of Applied Research and Technological Development, Fondazione IRCCS Istituto Nazionale dei Tumori, Milano, Italy; ^3^ Scientific Directorate, Azienda USL-IRCCS, Reggio Emilia, Italy; ^4^ Department of Rheumatology, Policlinico San Matteo IRCCS Fondazione, University of Pavia, Pavia, Italy; ^5^ Neuromuscular Diseases and Neuroimmunology Unit, Fondazione IRCCS Isitituto Neurologico Carlo Besta, Milano, Italy; ^6^ Humanitas Scientific Directorate, IRCCS Humanitas, Clinical and Research Center, Rozzano, Italy; ^7^ Department of Biomedical Sciences, Humanitas University, Milano, Italy; ^8^ William Harvey Research Institute, Queen Mary University, London, United Kingdom; ^9^ Unità di Reumatologia, Azienda unità sanitaria locale-IRCCS, Reggio Emilia, Italy; ^10^ Unità di Reumatologia, Università degli Studi di Modena e Reggio Emilia, Reggio Emilia, Italy; ^11^ Istituto di Ematologia “Seràgnoli” Azienda Ospedaliero-Universitaria di Bologna, IRCCS, Bologna, Italy; ^12^ Dipartimento di Medicina Specialistica, Diagnostica e Sperimentale Università di Bologna, Bologna, Italy; ^13^ Department of Neurosciences, Rehabilitation, Ophthalmology, Genetics, Maternal and Child Health and Center of Excellence for Biomedical Research, University of Genoa, Genoa, Italy; ^14^ IRCCS Ospedale Policlinico San Martino, Genoa, Italy; ^15^ University Vita-Salute San Raffaele, Milan, Italy; ^16^ Scientific Directorate, Fondazione IRCCS Istituto Nazionale dei Tumori di Milano, Milano, Italy; ^17^ Scientific Directorate, IRCCS Regina Elena, National Cancer Institute, Istituti Fisioterapici Ospitalieri (IFO), Rome, Italy; ^18^ Molecular Virology Unit, Fondazione IRCCS Policlinico San Matteo, Pavia, Italy; ^19^ Department of Clinical, Surgical, Diagnostics and Pediatric Sciences, University of Pavia, Pavia, Italy; ^20^ Scientific Directorate, San Gallicano Dermatological Institute IRCCS, Rome, Italy; ^21^ Department of Oncology and Hematology, Fondazione IRCCS Istituto Nazionale dei Tumori di Milano, Milano, Italy; ^22^ Department of Pediatric Hematology and Oncology and Cell and Gene Therapy, IRCCS Ospedale Pediatrico Bambino Gesù, Roma, Italy; ^23^ Department of Gynecology-Obstetrics and Pediatrics, University “La Sapienza”, Roma, Italy; ^24^ Cellular Immunology Laboratory, National Institute for Infectious Diseases L Spallanzani–IRCCS, Rome, Italy; ^25^ Infrastruttura Ricerca e Statistica, Azienda USL-IRCCS, Reggio Emilia, Italy; ^26^ Biostatistical Unit, Istituto Nazionale Tumori Regina Elena IRCCS-IFO, Rome, Italy; ^27^ Medical Oncology Department, IRCCS Istituto Tumori “Giovanni Paolo II”, Bari, Italy; ^28^ Department of Biomedical Sciences and Human Oncology, University of Bari “Aldo Moro”, Bari, Italy

**Keywords:** SARS-CoV-2 mRNA vaccine, safety, frail patients, COVID-19 infection, SARS-CoV-2 (COVID-19)

## Abstract

**Background:**

Frail patients are considered at relevant risk of complications due to coronavirus disease 2019 (COVID-19) infection and, for this reason, are prioritized candidates for vaccination. As these patients were originally not included in the registration trials, fear related to vaccine adverse events and disease worsening was one of the reasons for vaccine hesitancy. Herein, we report the safety profile of the prospective, multicenter, national VAX4FRAIL study (NCT04848493) to evaluate vaccines in a large trans-disease cohort of patients with solid or hematological malignancies and neurological and rheumatological diseases.

**Methods:**

Between March 3 and September 2, 2021, 566 patients were evaluable for safety endpoint: 105 received the mRNA-1273 vaccine and 461 the BNT162b2 vaccine. Frail patients were defined per protocol as patients under treatment with hematological malignancies (n = 131), solid tumors (n = 191), immune-rheumatological diseases (n = 86), and neurological diseases (n = 158), including multiple sclerosis and generalized myasthenia. The impact of the vaccination on the health status of patients was assessed through a questionnaire focused on the first week after each vaccine dose.

**Results:**

The most frequently reported moderate–severe adverse events were pain at the injection site (60.3% after the first dose, 55.4% after the second), fatigue (30.1%–41.7%), bone pain (27.4%–27.2%), and headache (11.8%–18.9%). Risk factors associated with the occurrence of severe symptoms after vaccine administration were identified through a multivariate logistic regression analysis: age was associated with severe fever presentation (younger patients vs. middle-aged vs. older ones), female individuals presented a higher probability of severe pain at the injection site, fatigue, headache, and bone pain; and the mRNA-1237 vaccine was associated with a higher probability of severe pain at the injection site and fever. After the first dose, patients presenting a severe symptom were at a relevant risk of recurrence of the same severe symptom after the second one. Overall, 11 patients (1.9%) after the first dose and 7 (1.2%) after the second one required postponement or suspension of the disease-specific treatment. Finally, two fatal events occurred among our 566 patients. These two events were considered unrelated to the vaccine.

**Conclusions:**

Our study reports that mRNA-COVID-19 vaccination is safe also in frail patients; as expected, side effects were manageable and had a minimum impact on patient care path.

## Introduction

The currently authorized messenger RNA (mRNA)-corona virus disease 2019 (COVID-19) vaccines—m-RNA-1237 Moderna (1) and BNT-162b2 Pfizer BioNTech (2)—have been evaluated in clinical trials that excluded, in accordance with the current regulations, immunocompromised subjects and restricted participation to healthy volunteers. Frail patients were not considered in such pivotal studies despite being the subjects at greatest risk of COVID-19 complications and with the potential greatest advantage.

Patients diagnosed with solid or hematological malignancy or under immunosuppressive treatment due to rheumatological or neurological diseases are considered at high risk of COVID-19 complications and are categorized as frail (3–7). The intended acceptance of the COVID-19 vaccine in frail patients confirms the positive attitudes towards vaccination, but questions arise around the safety of these vaccines in the setting of immune alterations engendered by their diseases and/or therapies (8–14). One of the most typical reasons for vaccine hesitancy has been fear related to vaccine side effects and underlying disease worsening. Of note, vaccine hesitancy has been reported also in the general population (vaccine acceptance up to 86.1% among healthcare students or 77.6% in the general population), as documented by large multinational studies (15–17), with concern about safety among the most given reasons to refuse vaccine.

Over the past 8 months, several groups have tried to answer questions about the efficacy and safety of COVID vaccination in different cohorts of frail subjects. No safety concerns emerged, confirming the profile described in the series of healthy subjects (14, 18–46) and pointing out an acceptable safety profile. VAX4FRAIL (47) study aimed at assessing immune responses to vaccination in a large trans-disease cohort of patients with hematological malignancies, solid tumors, and neurological and rheumatological diseases. The study’s main objective was to assess prospectively the immunological response to the COVID-19 vaccination in these specific subgroups, characterizing the kinetics of the immune response to the vaccination and its persistence over time. Longitudinal, prospective evaluation of the safety profile was part of this trans-disease study. Herein, we report safety profile results as outlined in VAX4FRAIL trial.

## Methods

Safety analysis was performed among patients enrolled in the VAX4FRAIL trial between t0 and t2 according to the protocol, t0 being the “time point 0” at first dose of vaccine and t2 the “time point 2” of the blood sampling 2–4 weeks after the second dose of vaccine (47). This is a national, multicentric observational prospective study conducted in Italy with the primary aim of assessing the immune response of COVID-19 vaccination in frail, immunocompromised patients.

Patients were considered for the current safety analysis if they met the general inclusion criteria: being ≥18 years of age, having received COVID-19 vaccination with mRNA vaccines (BNT-162b2 Pfizer-BioNTech or m-RNA-1237 Moderna vaccine), and having completed the health status assessment questionnaire after one of the two vaccine doses. Frail patients under evaluation were diagnosed with hematological malignancies, solid tumors, immune-rheumatological disease, and neurological disease. Detailed inclusion and exclusion criteria were previously reported (47), and disease stratification was conducted according to diagnosis and treatment of the primary disease.

The impact of the vaccination on the health status of patients was assessed through a questionnaire focused on the first week after each vaccine dose. We developed the questionnaire by listing the symptoms with the highest probability to be experienced by the patients. A 5-item Likert scale was derived from the Palliative Care Outcome Scale (POS). The POS is a questionnaire developed to assess the quality of life of patients with advanced disease. It was previously forward–backward translated from English, and it is validated both in English and in Italian (48).

The questionnaire was administered to the patients after the first vaccine dose (3–4 weeks after the first dose) and at the subsequent timing according to VAX4FRAIL protocol (2–4 weeks after the second dose). The questionnaire assessed how much the patient was troubled by eight symptoms (pain or swelling at the injection site, fatigue, headache, bone pain, fever, enlarged lymph nodes, skin rash, insomnia, diarrhea, and nausea or vomiting) (see **Supplementary Appendix 1**) The answers were graded according to a five-level scale (not at all, slightly, moderately, severely, and overwhelmingly). The patient was also asked to report and grade other symptoms possibly occurring after each vaccine dose and if he/she had to postpone or suspend therapies due to symptoms related to vaccination.

### Statistical Methods

The prevalence of symptoms after each dose of the vaccine was reported by aggregating the answers in three levels: score 1, no symptom (scored not at all); score 2, moderate symptom (scored slightly or moderately); and score 3, severe symptom (scored severely or overwhelmingly).

We estimated the probability of the occurrence of a severe symptom versus no symptom or moderate symptom after the second dose of the vaccine according to the occurrence of a severe symptom after the first dose using positive and negative predictive values (PPV and NPV, respectively). We also estimated the agreement between severe vs. non-severe symptom between the two doses using Cohen’s kappa statistics that adjust for the probability of the agreement occurring by chance. According to Landis and Koch, values <0 indicates no agreement, values between 0 and 0.20 as slight, 0.21–0.40 as fair, 0.41–0.60 as moderate, 0.61–0.80 as substantial, and 0.81–1 as almost perfect agreement (49).

The probability of occurrence of a severe symptom (scored 1) versus no symptom or moderate symptom (scored 0) after one of the two doses of the vaccine according to age, sex, main diagnosis, and type of vaccine was estimated in a multivariate logistic regression analysis adjusting for all variables.

The probability of occurrence of a severe symptom (scored 1) versus no symptom or moderate symptom (scored 0) after one of the two doses of the vaccine was estimated within each group of diseases by different subgroups and in a multivariate logistic regression analysis adjusting for age, sex, main diagnosis, and type of vaccine.

## Results

### Patients

**Figure 1:**
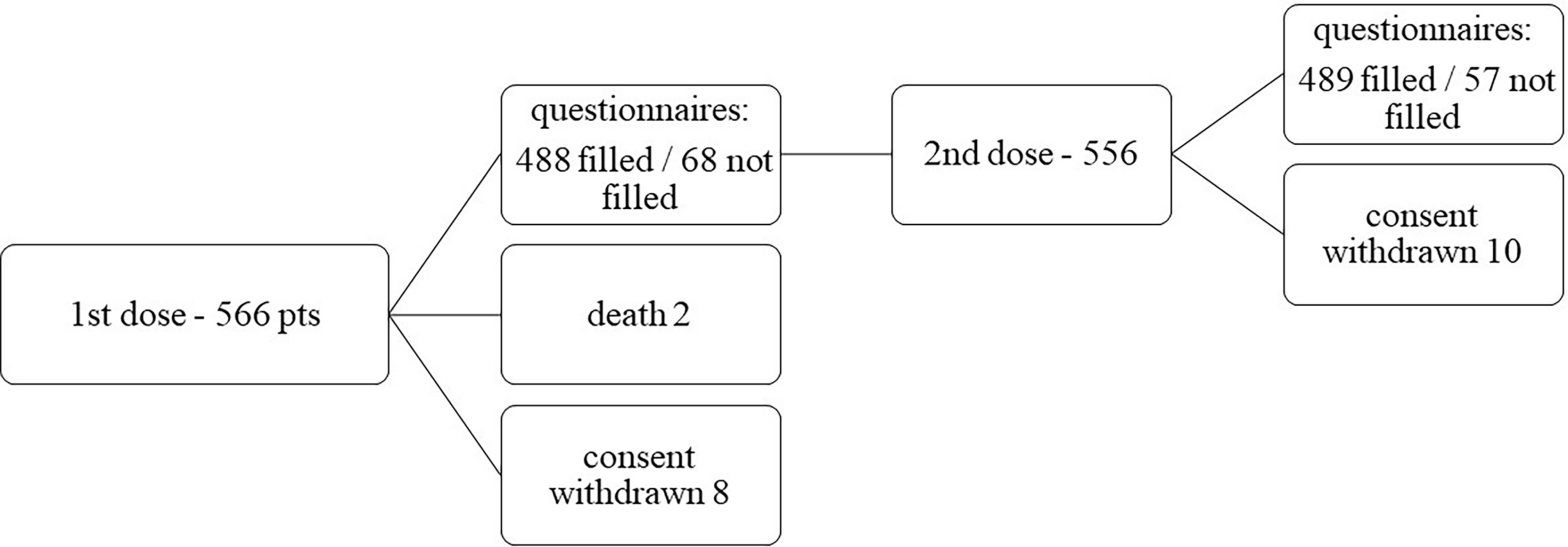
Patients’ disposition.

Between March 3 and September 2, 2021, 566 patients were enrolled in the VAX4FRAIL study and were eligible for this analysis. Among 566 patients who received the first dose of the mRNA vaccine, 105 received mRNA-1273, and 461 received BNT162b2. Among the 566 patients who received the first dose, 556 received the second one (**Figure 1**).

Overall, 488 patients after the first dose and 489 patients after the second dose were evaluable for safety evaluation having completed clinical evaluation and questionnaires. Sixty-eight patients after the first dose and 57 after the second dose did not fill the questionnaires. Consent withdrawn occurred in 8 cases after the first dose and 10 cases after the second one.

Patient characteristics are reported in **Table 1**. Most patients (N = 299, 52.8%) were aged between 51 and 70 years, and 317 (56.0%) were female. The cohort of patients with solid tumors comprised 191 patients (33.7%); the one with neurological diseases included multiple sclerosis and generalized myasthenia comprised 158 patients (27.9%). Overall, 131 patients were diagnosed with hematological malignancies (23.1%) and 86 with immunorheumathological diseases (15.2%).

**Table 1:** Patient’s characteristics.

	N (%)
**Age**	
18–50 years	160 (28.3)
51–70 years	299 (52.8)
>/= 71 years	107 (18.9)
**Patient sex**	
Male	249 (44.0)
Female	317 (56.0)
**Main diagnosis**	
Hematological malignancies	131 (23.1)
* New diagnosis*	30 (5.3)
* Chemotherapy (ongoing/completed <6 months)*	43 (7.6)
* Anti-B cells/Anti-CD30/Anti-PD1/CAR-T*	45 (8.0)
* Post transplant (auto/allo)*	13 (2.3)
Solid tumors	191 (33.7)
* Chemotherapy—adiuvant*	31 (5.5)
* Chemotherapy—metastatic disease*	73 (12.9)
* Immunotherapy—metastatic disease*	35 (6.2)
* Target therapy—metastatic disease*	52 (9.2)
Immunorheumatological diseases	86 (15.2)
* ANCA associated vasculitis—immunodepressants agents*	44 (7.8)
* ANCA associated vasculitis—Rituximab +/− steroids*	42 (7.4)
Neurological diseases	158 (27.9)
* Multiple Sclerosis*	58 (10.2)
* Generalized Myastenia Gravis*	100 (17.7)
**Vaccine**	
mRNA-1237 Moderna	105 (18.6)
BNT-162b2 Pfizer BioNTech	461 (81.4)
**All patients**	566 (100)

### Vaccine-Related Adverse Events

Overall, 438 (77.3%) patients reported any grade adverse events after the first dose (62, 11.0% reported as severe) and 373 (65.9%) after the second dose (87, 15.4% reported as severe). A detailed analysis of adverse events and severity is reported in **Table 2**.

**Table 2:** Occurrence of symptoms/adverse events over the week after vaccine administration.

	t1—One week after the first dose N = 488			t2—One week after the second dose N = 489			PPV (%)	NPV (%)	Kappa
	Not at all (%)	Moderate (%)	Severe (%)	Not at all (%)	Moderate (%)	Severe (%)			
*Pain*	194 (39.8)	263 (53.9)	31 (6.4)	218 (44.6)	246 (50.3)	25 (5.1)	17.2	96.2	0.15
*Fatigue*	341 (69.9)	125 (25.6)	22 (4.5)	285 (58.3)	171 (35.0)	33 (6.7)	28.6	93.9	0.18
*Headache*	430 (88.1)	48 (9.8)	10 (2.0)	397 (81.2)	78 (16.0)	14 (2.9)	25.0	97.7	0.18
*Pain bone*	408 (83.6)	67 (13.7)	13 (2.7)	356 (72.8)	114 (23.3)	19 (3.9)	25.0	96.8	0.18
*Fever*	459 (94.1)	23 (4.7)	6 (1.2)	411 (84.0)	49 (10.0)	29 (5.9)	83.5	94.8	0.28
*Enlarged lymph nodes*	487 (99.8)	–	1 (0.2)	478 (97.8)	10 (2.0)	1 (0.2)	NE	99.8	NE
*Skin rash*	471 (96.5)	14 (2.9)	3 (0.6)	458 (93.7)	22 (4.5)	9 (1.8)	NE	98.4	NE
*Insomnia*	464 (95.1)	22 (4.5)	2 (0.4)	443 (90.6)	41 (8.4)	5 (1.0)	NE	99.1	NE
*Diarrhea*	461 (94.5)	25 (5.1)	2 (0.4)	454 (92.8)	32 (6.5)	3 (0.6)	NE	99.6	NE
*Nausea or vomiting*	471 (96.5)	15 (3.1)	2 (0.4)	457 (93.5)	29 (5.9)	3 (0.6)	NE	99.6	NE

PPV (positive predictive value) probability of occurrence of a severe symptom after the second dose according to the occurrence of the same symptom after the first dose.

NPV (negative predictive value) probability of absence of a severe symptom after the second dose according to the not occurrence of the same after the first dose.

NE, not evaluable.

Kappa (Cohen’s kappa statistics) is a measure of agreement between severe vs. non-severe symptoms between the two doses.

“Moderate” and “Severe” include patients who have been troubled slightly or moderately (moderate) and severely or overwhelmingly (severe), respectively.

After the first dose (both mRNA-1273 and BNT162b2), 53.9% of the patients reported moderate pain at the injection site and 6.4% reported severe pain: this was the most frequently reported complaint after vaccine administration. Interestingly, after the second dose—t2—50.3% and 5.1% reported this adverse event as moderate and severe, respectively.

At t1, 25.6% of patients reported fatigue as a moderate event and 4.5% as a severe one. We observed a slight increase at t2, with 35% of patients reporting fatigue as a moderate event and 6.7% as a severe one.

Bone pain and headache were reported as moderate by 13.7% and 9.8% of patients at t1, while 2.7% and 2.0%, respectively, reported the symptoms as severe. After the second dose, bone pain was reported as moderate by 23.3% and severe by 3.9% of patients, while headache was reported as moderate by 16% and severe by 2.9% of patients.

Only 5.9% of patients reported fever as relevant symptoms at t1 (moderate 4.7%–severe 1.2%), while at t2, the percentage of patients reporting this symptom slightly increased: 15.9% overall, with 10.0% moderate and 5.9% severe. Only a minority of patients—<2%—reported nausea, diarrhea, insomnia, skin rash, or enlarged lymph nodes as severe manifestation both at t1 and t2.

Among unsolicited adverse events, after the first dose, 5 patients reported chills (5/5 moderate), 3 itching (2 moderate, 1 severe), 2 gastrointestinal pain (2/2 moderate), 5 myalgia (5/5 moderate), 6 dizziness (3 moderate, 3 severe), 1 drowsiness of moderate degree, and 2 sweats (1 patient moderate and 1 severe). Similarly, after the second dose, 12 patients reported chills (11 moderate, 1 severe), 7 itching (5 moderate, 2 severe), 1 a moderate gastrointestinal pain, 8 myalgia (6 moderate, 2 severe), 9 dizziness (8 moderate, 1 severe), 1 drowsiness of moderate degree, and 2 sweats (1 patient moderate and 1 severe). After the second dose, one patient reported confusion and one dysesthesia. Among the 11 patients with hematological malignancies who received an allogeneic stem cell transplantation, graft versus host disease (GvHD) occurrence or reactivation was not reported after the first and/or the second dose.

The agreement between the occurrence of a severe symptom after the doses (versus no symptom or a moderate symptom) was rather poor with a kappa statistic ranging between 0.15 and 0.28. Of note, the absence of severe symptoms after the first dose strongly predicts the high probability of the absence of the same severe symptoms after the second dose (**Table 2**). Patients reporting severe fever after the first dose were more prone to develop the same severe symptom after the second one.

### Multivariate Model for Occurrence of Severe Symptoms

A multivariate logistic regression—adjusted by age, sex, diagnosis, and vaccine—was performed to identify risk factors associated with the occurrence of severe symptoms after vaccine administration (**Table 3**).

**Table 3:** Probability of occurrence of a severe symptom after one of the two doses of vaccine estimated through a multivariate logistic regression adjusted by age, sex, diagnosis, and vaccine.

		Severe pain		Severe fatigue		Severe headache		Severe pain bone		Severe fever	
	N.	*N. (%)*	*Odds Ratio (95% CI)*	*N. (%)*	*Odds Ratio (95% CI)*	*N. (%)*	*Odds Ratio (95% CI)*	*N. (%)*	*Odds Ratio (95% CI)*	*N. (%)*	*Odds Ratio (95% CI)*
**Age**											
*18–50*	160	19 (11.9)	Ref.	17 (10.7)	Ref.	12 (7.5)	Ref	8 (5.0)	Ref	17 (10.7)	Ref
*51–70*	299	26 (8.7)	1.1 (0.6–2.2)	25 (8.4)	1.0 (0.5–2.0)	7 (2.3)	0.5 (0.2–1.3)	14 (4.7)	1.1 (0.4–2.8)	11 (3.7)	0.3 (0.1–0.7)
*71 +*	107	6 (5.6)	0.6 (0.2–1.6)	7 (6.5)	0.7 (0.3–1.9)	3 (2.8)	0.5 (0.1–2.0)	7 (6.5)	1.5 (0.5–4.4)	2 (1.9)	0.1 (0.1–0.7)
***p-value***		*0.401*		*0.740*		*0.315*		*0.725*		***0.003***	
**Sex**											
*Male*	249	10 (4.0)	Ref.	13 (5.2)	Ref.	2 (0.8)	Ref	7 (2.8)	Ref	9 (3.6)	Ref
*Female*	317	41 (12.9)	2.9 (1.4–6.2)	36 (11.4)	2.3 (1.2–4.5)	20 (6.3)	7.6 (1.7–33.6)	22 (6.9)	2.7 (1.1–6.5)	21 (6.6)	1.6 (0.7–3.8)
***p-value***		***0.005***		***0.016***		***0.007***		***0.028***		*0.253*	
**Diagnosis**											
*Hematological*	131	6 (4.6)	Ref.	9 (6.9)	Ref.	4 (3.1)	Ref	7 (5.3)	Ref	7 (5.3)	Ref
*Solid tumors*	191	4 (2.1)	0.3 (0.1–1.0)	10 (5.2)	0.7 (0.3–1.7)	2 (1.0)	0.2 (0.1–1.4)	8 (4.2)	0.7 (0–2–1.9)	10 (5.2)	0.8 (0.3–2.2)
*Rheumatological*	86	15 (17.4)	4.0 (1.4–11.0)	10 (11.6)	1.5 (0.6–4.0)	3 (3.5)	0.8 (0.2–3.7)	4 (4.7)	0–7 (0.2–2.6)	5 (5.8)	1.0 (0.3–3.5)
*Neurological*	158	26 (16.5)	2.8 (1.1–7.5)	20 (12.7)	1.7 (0.7–4.1)	13 (8.2)	1.7 (0.5–5.8)	10 (6.3)	1.1 (0.4–3.0)	8 (5.1)	0.5 (0.2–1.6)
***p-value***		***<0.001***		*0.108*		*0.090*		*0.761*		*0.646*	
**Vaccine**											
*m-RNA-1237*	105	19 (18.1)	Ref.	9 (8.6)	Ref.	6 (5.7)	Ref	6 (5.7)	Ref	12 (11.4)	Ref
*BNT-162b2*	461	32 (6.9)	0.3 (0.1–0.6)	40 (8.7)	1.1 (0.5–2.4)	16 (3.5)	0.7 (0.3–2.0)	23 (5.0)	0.9 (0.4–2.5)	18 (3.9)	0.3 (0.1–0.7)
***p-value***		***0.001***		*0.826*		*0.510*		*0.923*		***0.004***	

Ref. is the referent strata.

The p-values in bold underline the statistically significant results.

Age was associated with severe fever presentation: younger patients (age 18–50 years) had a higher probability of occurrence of severe fever than middle-aged patients [age 51–70; odds ratio, 0.3; 95% confidence interval (CI), 0.1–0.7] or older ones (>/= 71; odds ratio, 0.1; 95% CI, 0.1–0.7)—p = 0.003.

Female patients presented higher probability of severe pain at the injection site (odds ratio, 2.9; 95% CI, 1.4–6.2; p = 0.005), severe fatigue (odds ratio, 2.3; 95% CI, 1.2–4.5; p = 0.016), severe headache (odds ratio, 7.6; 95%, 1.7–33.6, p = 0.007), and severe bone pain (odds ratio, 2.7; 95% CI, 1.1–6.5; p = 0.028).

Diagnosis (hematological diseases, solid tumors, neurological diseases, and rheumatological diseases) has no impact on the occurrence of severe fatigue, headache, bone pain, or fever but was associated with severe pain at the injection site: patients with a diagnosis of solid tumors reported severe pain less frequently than patients with hematological diseases (odds ratio, 0.3; 95% CI, 0.1–1.0), patients with a diagnosis of neurological or rheumatological disease have a higher probability of occurrence of severe pain at the injection site in comparison with patients with hematological disease (odds ratio for neurological diseases, 4; 95% CI, 1.4–11; the odds ratio for rheumatological diseases, 2.8; 95% CI, 1.1–7.5)—p < 0.001.

The probability of occurrence of a severe symptom versus no symptom or moderate symptom after one of the two doses of the vaccine was estimated within each group of diseases by different subgroups and in an adjusted multivariate logistic regression analysis (**Supplementary Table 1**). Notwithstanding that the only statistically significant differences were reported in the solid tumor cohort for the symptoms severe fatigue and headache, the small number of patients in each subgroup and the lack of correction for multiplicity make it necessary to consider these results with caution.

Finally, the conditional contribution made by the vaccine (mRNA-1237 versus BNT162b2) clearly showed that mRNA-1237 was associated with a higher probability of severe pain at the injection site (odds ratio, 0.3; 95% CI, 0.1–0.6; p = 0.001) and a higher probability of severe fever (odds ratio, 0.3; 95% CI, 0.1–0.7; p = 0.004).

### Impact on Disease and Therapy

Overall, 11 patients (1.9%) after the first dose and 7 (1.2%) after the second dose required postponement or suspension of the disease-specific treatment due to the occurrence of symptoms associated with the vaccine. Among the 11 patients who required delays after the first dose, 7 were diagnosed with hematological disease and 4 with a solid tumor. Among the seven patients who required delays after the second dose, three were diagnosed with the hematological disease and four with a solid tumor.

No patients from the neurological diseases’ cohort or the rheumatological diseases’ cohort required treatment delay.

Fatal events, long-term sequelae, or hospitalization related to the vaccine was not registered. Two patients died from disease progression; the events were clearly reported by the investigator as not related to the vaccine administration.

## Discussion

Questionnaires based upon open and/or closed questions administered through the web-based app, phone interview, self-reporting forms, and face-to-face visits were all utilized to collect adverse events after COVID-19 vaccinations in the effort to clarify the safety of vaccines better. Absence of a univocal method to grade and collect this information makes it almost impossible to directly compare the different experiences, but, of note, all the studies—both retrospective and prospective—are concordant in underlying the general perception of the absence of safety issue among distinct cohorts of frail patients (**Table 4**).

**Table 4:** Literature review.

Cohort	N of pts	Control group	Vaccine	Methods for evaluation of AE	AE reported	Severe AE
**Allo-HSCT** **(** 20, 24 **)**	192	26 healthy subjects	BNT162b2	Questionnaire within 7 days following the first vaccination (24). Interview and clinical evaluation, grading CTC AE v5 (20)	Only grade 1 or 2 AEs were observed. Cytopenia 12% after the 1st dose and 10% after the 2nd dose (20). 3 cases of GvHD exacerbation after each dose (20).	There were no grade 3 or 4 non-hematological adverse events. A single case of impending graft rejection was summarized as possibly related.
**Oncohematology (** 23, 25 **)**	977	67 healthy subjects + 36 elderly w/o cancers	BNT162b2	Questionnaires adapted from the original phase 3 BNT162b2 trial (25) Registration of local and systemic side effects (23)	AEs were more common after the 2nd dose (25). Mild pain at the injection site most common side effect in all the cohorts (23).	No grade 4 adverse events were reported
**Cancer (** 19, 21, 38–42, 44, 45 **)**	2,387	739 healthy subjects	BNT162b2 mRNA-1273 ChAdOx1	Solicited and unsolicited AE collection through face-to-face or telephone consultations (19). Weekly telephone consultation (21). Patient-reported outcomes between 1–2–4 weeks after each dose (38). Web-based electronic platform for home toxicity monitoring (39) AE graded according to patients interview within 2–4-week windows (40). Patient-reported scale (very mild/mild/moderate/severe) (41). Questionnaire (42, 44). Review of medical records (45).	The most frequently reported local AE was mild-to-moderate pain at the injection site (39). AEs occurred between 39% and 56% patients after the 1^st^ dose and between 29% and 58% after the 2^nd^ (21, 41, 44) 1st dose AEs: local side effects 29.8%–31.5%, fatigue 8.3%–7%, headaches 3.9%–8.17% and arthralgia/myalgia 3.7%–2.71% (38–41). 2nd dose AEs: fever 5.8%–14.1%, local reactions 12.6%–33.46%, fatigue 10.4%–8.9%, arthralgia/myalgia 5.2% (41). Overall: 45%–69% injection site pain, myalgia 9.5%–15%, bone pain 5%, headache 10%–24.3%, fatigue 10%–28.6%, chills 10%, appetite loss 5% (19, 40, 42, 45). AEs were more common after the 2nd vaccine dose than that with the 1st (33.3% vs. 7%) (44).	No grade 3/4 side effects were recorded (21, 38, 40, 42, 44, 45). Low rate of grade 3/4 AE: 2.1% vaccine related AE grade 3 (19), 3.3% of patients after the 1st inoculum and 1.4% after the booster (41). One sudden death occurred after 5 days of the vaccine in a hospitalized patient who received active chemotherapy for advanced bladder cancer. The post-mortem examination revealed pulmonary embolism (41). Three cancer-related deaths were considered unrelated to the BNT162b2 vaccine (39). One patient died due to myocardial infarction—the authors cannot complete rule out that this event was vaccine related (39). Severe reactogenicity occurred in 1% of the patients following the priming dose and in 3% of the patients following the booster dose (39).
**Cancers** **(checkpoint inhibitors) (** 18, 43 **)**	222	2,241 healthy subjects	BNT162b2	Solicited and unsolicited AE collection Telephone questionnaires 17–21 days after the first vaccine dose and median 19 days after the second	1st dose AEs pain at the injection site 21%–28.57% (18)– (43). 2nd dose AEs fever 6.94% (43,) pain at the injection site 63%, local rash 2%, local swelling 9%; muscle pain 34%, fatigue 34%, headache 16%, fever 10%, chills 10%, gastrointestinal complications 10%, flu-like symptoms 2% (18). Systemic events more frequent after the 2nd dose, while local effects were less common (43).	No thrombosis, hypersensitivity adverse events or vaccine-related anaphylaxis were signaled. One patient has reported two immune-related side effects (hepatitis G3 and colitis G3) 10 days after the first dose of vaccine.
**Multiple sclerosis** **(** 26–28, 37 **)**	896	7 healthy subjects	BNT162b2 ChAdOx1 mRNA-1273	Questionnaires (26, 28) Telemedicine and face-to-face evaluation (7–21 days after each dose) (27)	Between 56.9% and 94% reported AEs (26, 28). AEs: sore arm 70%, flu-like symptoms 64%, fever 21%, fatigue 27%, headache 21% (27, 28, 37).	No severe adverse effects occurred (28) 15.1% of participants reported new or worsening neurological symptoms (26). Acute relapse 2.1% and 1.6% following the first and second doses, respectively (27). 2 people reported new or worsening MS symptoms (37)
**Systemic autoimmune Disease** **(** 14, 22, 29–36, 46 **)**	6492	394 healthy subjects + 26 patients COVID recovered (28)	BNT162b2 ChAdOx1 mRNA-1273 BBV152 CoronaVac Ad26COV2.S	Registration of local and systemic side effects (22) Questionnaire distributed *via* e-mail (35), phone (29), web-based (32) 7th days after vaccination collection of data (33) Questionnaire within the 1st week after vaccination (34) Questionnaires, (30, 31, 36, 46)	Absence of AEs 20% of patients (22). Overall occurrence of AEs 35%–60.5% (14, 33, 35, 36, 46) AEs: local reactions 17%–89%; systemic symptoms 69%, headache 12%–20%; muscle sore 10%–22.8% fatigue 7.4%–33.4% (22, 30, 31, 33, 36). 1st dose AEs 45%–53%, 2nd dose AEs 26%–53% (30, 32). Patients who received both vaccine doses and reported side effects after the 1st dose were more likely to report side effects after the 2nd dose than those who did not (relative risk 2·30, 95% CI 1·88–2·82; p < 0.0001) (32).	No participants reported severe adverse events (22, 32–34, 46). Severe adverse events 1% (35). Major adverse events: death (2) several weeks after the 2nd dose, non-disseminated herpes zoster (6), uveitis (2), and pericarditis (1) (29). Postvaccination disease activity remained stable in the majority of patients (29). Relapses of the underlying disease 2.2% of patients after the first dose of vaccine (30). 11% of patients reported that their psoriasis worsened after vaccination (22). Immunosuppressive treatment was postponed because of COVID-19 vaccination in 6% patients (35) Four patients reported flare of arthritis that resolved within 5 days (33). One patient with Familial Mediterranean fever reported new-onset arthritis 2 weeks after the 1st dose. No flare-up of the underlying IRD occurred in any other patient (31). Flares of existing systemic rheumatic disease 13.4%, 4.6% requiring treatment (36).

Providing information on the safety of COVID-19 vaccination in high-risk frail populations is a duty of the international scientific community. To date, several groups are trying to answer two fundamental questions: (i) if and how frail patients develop an effective response to the vaccine and (ii) if the safety profile is confirmed valid in this category both with reference to toxicity and with reference to the maintenance of control of the underlying disease.

Knowledge of COVID-19 vaccine-related side effects have a crucial role in the public decision regarding vaccination. Studies designed to monitor the safety and effectiveness of COVID-19 vaccines globally are ongoing, focusing on short-term side effects, the booster doses’ side effects, and the long-term safety and effectiveness (50).

Safety profile is confirmed acceptable in all the experience reported so far in frail patients.

More systemic and local side effects were observed after the second dose of vaccine than after the first dose. The most common local side effects were pain at the injection site, local rash, and local swelling, whereas the most common systemic side effects were muscle pain, fatigue, headache, fever, chills, gastrointestinal complications, and flu-like symptoms (18, 19, 22–24, 26–28, 30, 31, 36–38). Overall, the incidence of severe symptoms was low—<2.5% (19, 39, 41)—with authors reporting no severe grade 3–4 adverse events (20–22, 24, 33, 38, 42–46). In a national prospective cohort study evaluating outcomes in patients with hematological malignancies in Lithuania (25), the authors reported that adverse events were more common after the second dose, with fatigue being the most prevalent symptom (13%). No grade 4 adverse events were reported.

Among patients with multiple sclerosis, Lotan and colleagues (26) outlined how 15% of the participants reported new or worsening neurological symptoms following the vaccination, the most frequent being sensory disturbances (58.3%). Most symptoms occurred within the first 24 h after vaccination and resolved within 3 days. A total of 28 participants (77.8%) did not require any medication to treat their symptoms. Of note, no increased risk of relapse activity was noted across patients with multiple sclerosis (26, 27) or autoimmune inflammatory rheumatic diseases (29–31): Boekel and coworkers clearly showed how multivariable logistic regression analyses showed similar odds for any adverse event, systemic adverse events, or moderate or severe adverse events between patients and controls, which was consistent when patients with rheumatoid arthritis or multiple sclerosis were compared with healthy controls (36).

In our experience, cohort composition was clearly defined at enrolment of patients, identifying a subset of high-risk subjects for severe COVID-19. The monitoring strategy was defined per protocol and homogeneous across the four categories of diseases (hematological disease, solid tumors, neurological conditions, and rheumatological diseases).

Overall, our study confirms the positive safety profile reported by other authors in both prospective and retrospective analyses (**Table 4**). Incidence of severe adverse events after vaccine administration was generally low and <3%. Most frequent complaints were pain at the injection site (severe, 6.3%) and fatigue (severe, 4.5%) after the first dose; pain at the injection site (severe, 5.1%), fatigue (severe, 6.8%), bone pain (severe, 3.9%) and fever (severe, 6%) after the second dose.

Patients experiencing a severe symptom after the first dose were more likely to report the same after the second one. Similarly, the absence of a severe symptom after the first dose was significantly associated with an absence of the same symptoms also after the second dose. This observation should be taken into consideration during the counseling of patients: patients should be reassured about the possibility of adopting a preventive strategy to reduce the burden of symptoms and on the not-unexpected onset of the symptom itself.

Moreover, the definition of predisposing factors to an increased possibility of presenting specific adverse events (e.g., younger patients are more likely to experience fever) can help both in the implementation of preventive measures (e.g., pre-emptive administration of painkiller drugs or antifever), in the optimization of counseling (e.g., if a patient experienced adverse events after the first shot, he should be advised that the recurrence of the same after the second is very likely and that this is not unexpected; moreover, strategies to counterbalance this inconvenience can be applied), and in the planning of therapies and/or vaccination itself.

According to the design of the study, the patient population was selected for the intrinsic immunocompromised condition—related both to the underling disease and the given treatments. Very few patients in each subgroup experienced severe symptoms, and this makes difficult to draw any additional conclusion (e.g., correlation between disease–treatment–disease status and occurrence of severe adverse events). Pursuing the monitoring of frail patients will further enlighten possible influencing factors.

Of note, <2% of patients required a delay or a suspension of the ongoing or planned treatment of the underlying diseases due to vaccination. This pointed out the positive safety profile of the vaccine strategy; furthermore, this aspect should be discussed with patients to confirm the absence of impact on the whole therapy program. Whatever the underlying disease (a hematological cancer, a solid tumor, a rheumatological disease, or a neurological disease), the mRNA-COVID-19 vaccine should be considered a crucial step to allow a safe program of treatment more than a possible obstacle or danger to pursue the control of the disease, being the safety profile reassuring. Fatal events or hospitalization related to the vaccine were not registered in our cohort of high-risk frail patients.

Despite the absence of safety concerns emerging from our study, we suggest that it is mandatory to maintain constant and careful surveillance concerning post-vaccination adverse events. This can only contribute to the reliability that the vaccination strategy pursues and represents a cornerstone of general medical practice.

As outlined by several authors (3–9), one of the most common reasons for vaccine hesitancy was fear related to vaccine side effects and disease worsening: we can confirm that vaccine side effects were both manageable and in line with previously reported in the general population, without the occurrence of unexpected events and no concern related to worsening of the underlying disease or the need to delay treatment.

To further strengthen the safety of the vaccine in frail patients, we underline that our safety results are in line with what has been observed in the pivotal studies conducted in immunocompetent subjects. Safety assessments included monitoring of solicited local and systemic adverse events for 7 days after each injection for both mRNA-1273 (1) and BNT162b2 vaccine (2).

In the mRNA-1273 (1) group, the adverse events at the injection site (pain was the most common event) occurred after the first dose in 84.2% of subjects, and the severity was mainly grade 1 or 2. After the first dose, 54.9% of subjects reported systemic adverse events; after the second dose, this percentage increased up to 79.4%, and, similarly, the severity increased. Authors reported that both solicited injection site and systemic adverse events were more common among younger participants (18–<65 years of age) than among older participants (≥65 years of age).

Similarly, in the BNT162b2 (2) group, local reactogenicity (pain at the injection site) was reported frequently (85% of subjects <55 years and 71% of >55 years after the first dose, 78% and 66% respectively, after the second). The proportion of participants reporting local reactions did not increase after the second dose, and no participant reported a grade 4 local reaction. In general, local reactions were mostly mild to moderate in severity. Systemic events were reported more often by younger vaccine recipients (16–55 years of age) than by older vaccine recipients (more than 55 years of age) and more often after the second dose than the first dose. The most reported systemic events were fatigue and headache.

## Conclusion

Frail patients who are candidates to mRNA-COVID-19 vaccination should be reassured about the safety profile of vaccine strategy: adverse events were in line with the report from the healthy cohort of subjects and national observatories, no evidence of worsening of the underlying disease was reported, and no concern on the adherence to the treatment program of the disease itself emerged from our prospective multicenter national study. Pursuing careful and timely monitoring of expected and unexpected adverse events represents a gold standard of modern medicine and can only support evidence-based medicine.

## Data Availability Statement

All data produced in the present study are available upon reasonable request to the authors.

## Ethics Statement

The protocol has been approved by national competent authorities (AIFA) and the ethics committee of the National Institute for Infectious Diseases Lazzaro Spallanzani (IRCCS). The patients/participants provided their written informed consent to participate in this study.

## The VAX4FRAIL Study Group:

**Principal Investigators** (alphabetical order): Giovanni Apolone (Fondazione IRCCS Istituto Nazionale dei Tumori di Milano); Alberto Mantovani (IRCCS Istituto Clinico Humanitas, Milano).

**Scientific Coordinator**: Massimo Costantini (Fondazione IRCCS Istituto Nazionale dei Tumori di Milano).

**Steering Committee** (alphabetical order): Chiara Agrati (IRCCS Istituto per le Malattie Infettive Lazzaro Spallanzani, Roma); Giovanni Apolone (Fondazione IRCCS Istituto Nazionale dei Tumori di Milano); Fabio Ciceri (IRCCS Ospedale San Raffaele, Milano); Gennaro Ciliberto (IRCCS Istituto Nazionale Tumori Regina Elena, Roma); Massimo Costantini (Fondazione IRCCS Istituto Nazionale dei Tumori di Milano); Franco Locatelli (Università La Sapienza, Roma); Alberto Mantovani (IRCCS Istituto Clinico Humanitas, Milano); Fausto Baldanti (Fondazione IRCCS Policlinico San Matteo di Pavia); Aldo Morrone (Istituto Dermatologico San Gallicano IRCCS, Roma); Carlo Salvarani (Azienda USL-IRCCS Reggio Emilia); Nicola Silvestris (IRCCS Istituto Tumori “Giovanni Paolo II,” Bari); Fabrizio Tagliavini (Fondazione IRCCS Istituto Neurologico Carlo Besta, Milano); Antonio Uccelli (Ospedale Policlinico San Martino IRCCS, Genova); Pier Luigi Zinzani (IRCCS Azienda Ospedaliero-Universitaria di Bologna).

**Disease Groups**

1. Hematological Malignancies Referent: Paolo Corradini (Fondazione IRCCS Istituto Nazionale dei Tumori, Milano);
2. Solid Tumors Referent: Gennaro Ciliberto (IRCCS Istituto Nazionale Tumori Regina Elena, Roma);
3. Immunorheumatological Diseases Referent: Carlo Salvarani (Azienda USL IRCCS Reggio Emilia);
4. Neurological Diseases: Referent: Antonio Uccelli (Ospedale Policlinico San Martino IRCCS, Genova); Renato Mantegazza (Fondazione I.R.C.C.S Istituto Neurologico Carlo Besta (INCB), Milano).

## Immunological Group

Referents: Chiara Agrati (IRCCS Istituto per le Malattie Infettive Lazzaro Spallanzani, Roma); Maria Rescigno (IRCCS Istituto Clinico Humanitas, Milano); Daniela Fenoglio (Ospedale Policlinico San Martino IRCCS, Genova);

Participants: Roberta Mortarini (Fondazione IRCCS Istituto Nazionale dei Tumori di Milano); Cristina Tresoldi (IRCCS Ospedale San Raffaele, Milano); Laura Conti (IRCCS Istituto Nazionale.

Tumori Regina Elena, Roma); Stefania Croci (Azienda USL IRCCS Reggio Emilia); Fausto Baldanti (Fondazione IRCCS Policlinico San Matteo di Pavia); Vito Garrisi (IRCCS Istituto Tumori “Giovanni Paolo II,” Bari); Fulvio Baggi (Fondazione IRCCS Istituto Neurologico Carlo Besta, Milano); Tiziana Lazzarotto (IRCCS Azienda Ospedaliero-Universitaria di Bologna); Fulvia Pimpinelli (Istituto Dermatologico San Gallicano IRCCS, Roma).

## INMI Centralized Laboratory (INMI Lazzaro Spallanzani–IRCCS, Roma) (alphabetical order)

Enrico Girardi (Scientific Director), Aurora Bettini; Veronica Bordoni; Concetta Castilletti; Eleonora Cimini; Rita Casetti; Francesca Colavita; Flavia Cristofanelli; Massimo Francalancia; Simona Gili; Giulia Gramigna; Germana Grassi; Daniele Lapa; Sara Leone; Davide Mariotti; Giulia Matusali; Silvia Meschi; Stefania Notari; Enzo Puro; Marika Rubino; Alessandra Sacchi; Eleonora Tartaglia

## Clinical Task Force

Paolo Corradini, Silvia Damian, Filippo de Braud (Fondazione IRCCS Istituto Nazionale dei Tumori di Milano); Maria Teresa Lupo-Stanghellini, Lorenzo Dagna, Francesca Ogliari, Massimo Filippi (IRCCS Ospedale San Raffaele: Milano); Giulia Piaggio (IRCCS Istituto Nazionale Tumori Regina Elena, Roma); Elena Azzolini, Chiara Pozzi, Luca Germagnoli, Carlo Selmi, Maria De Santis, Carmelo Carlo-Stella, Alexia Bertuzzi, Francesca Motta, Angela Ceribelli (IRCCS Istituto Clinico Humanitas, Milano);

Fausto Baldanti, Sara Monti (Fondazione IRCCS Policlinico San Matteo di Pavia); Aldo Morrone (Istituto Dermatologico San Gallicano IRCCS, Roma); Maria Grazia Catanoso, Carmine Pinto, Francesco Merli, Franco Valzania, Monica Guberti (Azienda USL-IRCCS Reggio Emilia); 

Rosa Divella, Antonio Tufaro, Vito Garrisi, Sabina Delcuratolo, Mariana Miano (IRCCS Istituto Tumori “Giovanni Paolo II,” Bari);

Carlo Antozzi, Silvia Bonanno Rita Frangiamore, Lorenzo Maggi (Fondazione IRCCS Istituto Neurologico Carlo Besta, Milano); Antonio Uccelli, Paolo Pronzato, Matilde Inglese, Carlo Genova, Caterina Lapucci, Alice Laroni, Ilaria Poirè (Ospedale Policlinico San Martino IRCCS, Genova);

Marco Fusconi, Vittorio Stefoni, Maria Abbondanza Pantaleo (IRCCS Azienda Ospedaliero-Universitaria di Bologna).

## Statistical Committee

Diana Giannarelli (IRCCS Istituto Nazionale Tumori Regina Elena, Roma).

## e-CRF and Monitoring Referent:

Valentina Sinno, Serena Di Cosimo (Fondazione IRCCS Istituto Nazionale dei Tumori di Milano).

## Project Managers of the Study:

Referents: Elena Turola, Azienda USL-IRCCS di Reggio Emilia.

Participants: Iolanda Pulice, Roberta Mennitto Fondazione IRCCS Istituto Nazionale dei Tumori, Milano); Stefania Trinca (IRCCS Ospedale San Raffaele, Milano); Giulia Piaggio (IRCCS Istituto Nazionale Tumori Regina Elena, Roma); Chiara Pozzi (IRCCS Istituto Clinico Humanitas, Milano);

Irene Cassaniti (Fondazione IRCCS Policlinico San Matteo, Pavia); Alessandro Barberini (Istituto Dermatologico San Gallicano IRCCS, Roma); Arianna Belvedere (Azienda USL-IRCCS Reggio Emilia);

Sabina Del Curatolo (IRCCS Istituto Tumori “Giovanni Paolo II,” Bari); Rinaldi Elena, Federica Bortone (Fondazione IRCCS Istituto Neurologico Carlo Besta, Milano); Maria Giovanna Dal Bello (Ospedale Policlinico San Martino IRCCS, Genova); Silvia Corazza (IRCCS Azienda Ospedaliero-Universitaria, Bologna).

## Author Contributions

MC, VS, and ET had full access to all the data in the study and took responsibility for the integrity of the data and the accuracy of the data analysis. Concept and design: MTLS, SDC, MC, SM, NS, GA, and AMa. Acquisition, analysis, or interpretation of data: MTLS, SDC, MC, SM, and NS. Drafting of the manuscript: MTLS, SDC, SM, MC, and NS. Critical revision of the manuscript for important intellectual content: all authors. Statistical analysis: MC, DG, and ET. Administrative, technical, or material support: VS and ET. All authors contributed to the article and approved the submitted version.

## Funding

This study has been financed by Italian Ministry of Health within Ricerca Corrente 2021-Special Projects-Vax4Frail.

## Conflict of Interest

The authors declare that the research was conducted in the absence of any commercial or financial relationships that could be construed as a potential conflict of interest.

## Publisher’s Note

All claims expressed in this article are solely those of the authors and do not necessarily represent those of their affiliated organizations, or those of the publisher, the editors and the reviewers. Any product that may be evaluated in this article, or claim that may be made by its manufacturer, is not guaranteed or endorsed by the publisher.
