# Supplementary material for: mRNA-COVID19 Vaccination Can Be Considered Safe and Tolerable for Frail Patients
Source: Front Oncol. 2022 Mar 17;12:855723. doi: 10.3389/fonc.2022.855723 (PMC8969577; doi:10.3389/fonc.2022.855723)
Supplement: Supplementary file 2 [file DataSheet_2.docx]

**Supplementary Table 1** - Probability of occurrence of a severe symptom after one of the two doses of vaccine

|  |  | **Severe pain** | **Severe fatigue** | **Severe headache** | **Severe pain bone** | **Severe fever** |
| --- | --- | --- | --- | --- | --- | --- |
|  | N. | N. (%) | N. (%) | N. (%) | N. (%) | N. (%) |
| **Haematological malignancies (total)** |  |  |  |  |  |  |
| New diagnosis | 30 | 1 (3.3) | - | - | - | - |
| Chemotherapy (ongoing or completed <6 months) | 43 | 3 (7.0) | 2 (4.7) | 2 (4.7) | 3 (7.0) | 2 (4.7) |
| Anti-B cells or Anti-CD30 or Anti-PD1 or CAR-T | 45 | 1 (2.2) | 6 (13.3) | 2 (4.4) | 4 (8.9) | 3 (6.7) |
| Post-transplant (auto or allo) | 13 | 1 (7.7) | 1 (7.7) | - | - | 2 (15.4) |
|  |  | P=0.675 | P=0.139 | P=0.568 | P=0.287 | P=0.214 |
| **Solid tumours** |  |  |  |  |  |  |
| Adjuvant Chemotherapy | 31 | - | 5 (16.1) | - | 1 (3.2) | 3 (9.7) |
| Chemotherapy for metastatic disease | 73 | 2 (2.7) | 1 (1.4) | - | 2 (2.7) | 3 (4.1) |
| Immunotherapy for metastatic disease | 35 | - | 2 (5.7) | 2 (5.7) | 3 (8.6) | 2 (5.7) |
| Target therapy for metastatic disease | 52 | 2 (3.8) | 2 (3.8) | - | 2 (3.8) | 2 (3.8) |
|  |  | P=0.505 | P=0.020 | P=0.029 | P=0.543 | P=0.651 |
| **Immunorheumatological diseases** |  |  |  |  |  |  |
| ANCA associated vasculitis (Immunodepressants agents) | 44 | 7 (15.9) | 6(13.6) | 2 (4.5) | 2 (4.5) | 2 (4.5) |
| ANCA associated vasculitis (Rituximab +/- steroids) | 42 | 8 (19.0) | 4 (9.5) | 1 (2.4) | 2 (4.8) | 3 (7.1) |
|  |  | P=0.701 | P=0.552 | P=0.584 | P=0.962 | P=0.607 |
| **Neurological diseases** |  |  |  |  |  |  |
| Multiple Sclerosis | 58 | 8 (13.8) | 9 (15.5) | 5 (8.6) | 5 (8.6) | 4 (6.9) |
| Generalized Myasthenia Gravis | 100 | 18 (18.0) | 11 (11.0) | 8 (8.0) | 5 (5.0) | 4 (4.0) |
|  |  | P=0.492 | P=0.410 | P=0.891 | P=0.368 | P=0.423 |
